# Decline in temperature and humidity increases the occurrence of influenza in cold climate

**DOI:** 10.1186/1476-069X-13-22

**Published:** 2014-03-28

**Authors:** Kari Jaakkola, Annika Saukkoriipi, Jari Jokelainen, Raija Juvonen, Jaana Kauppila, Olli Vainio, Thedi Ziegler, Esa Rönkkö, Jouni JK Jaakkola, Tiina M Ikäheimo

**Affiliations:** 1 Centre for Military Medicine, the Finnish Defence Forces, P.O. Box 2, FI-17701 Lahti, Finland; 2 Department of Vaccinations and Immune Protection, National Institute for Health and Welfare, P.O. Box 310, FI-90101 Oulu, Finland; 3 Institute of Health Sciences, University of Oulu, P.O. Box 5000, FI-90014 Oulu, Finland; 4 Unit of General Practice, Oulu University Hospital, P.O. Box 20, FI-90029 Oulu, Finland; 5 Department of Otorhinolaryngology, Kainuu Central Hospital, Sotkamontie 13, FI-87140 Kajaani, Finland; 6 Northern Finland Laboratory Centre (NordLab), Oulu, Finland; 7 Institute of Diagnostics, Department of Microbiology and Immunology, University of Oulu, P.O. Box 5000, FI-90014 Oulu, Finland; 8 Department of Infectious Disease Surveillance and Control, National Influenza Center, National Institute for Health and Welfare, P.O. Box 30, FI-00271 Helsinki, Finland; 9 Center for Environmental and Respiratory Health Research, University of Oulu, P.O. Box 5000, FI-90014 Oulu, Finland; 10 Respiratory Medicine Unit, Department of Medicine, Oulu University Hospital, P.O.Box 20, FI-90029 Oulu, Finland; 11 Medical Research Center Oulu, Oulu, Finland

**Keywords:** Influenza, Low temperature, Absolute humidity

## Abstract

**Background:**

Both temperature and humidity may independently or jointly contribute to the risk of influenza infections. We examined the relations between the level and decrease of temperature, humidity and the risk of influenza A and B virus infections in a subarctic climate.

**Methods:**

We conducted a case-crossover study among military conscripts (n = 892) seeking medical attention due to respiratory symptoms during their military training period and identified 66 influenza A and B cases by PCR or serology. Meteorological data such as measures of average and decline in ambient temperature and absolute humidity (AH) during the three preceding days of the onset (hazard period) and two reference periods, prior and after the onset were obtained.

**Results:**

The average temperature preceding the influenza onset was −6.8 ± 5.6°C and AH 3.1 ± 1.3 g/m^3^. A decrease in both temperature and AH during the hazard period increased the occurrence of influenza so that a 1°C decrease in temperature and 0.5 g decrease per m^3^ in AH increased the estimated risk by 11% [OR 1.11 (1.03 to 1.20)] and 58% [OR 1.58 (1.28 to 1.96)], respectively. The occurrence of influenza infections was positively associated with both the average temperature [OR 1.10 per 1°C (95% confidence interval 1.02 to 1.19)] and AH [OR 1.25 per g/m^3^ (1.05 to 1.49)] during the hazard period prior to onset.

**Conclusion:**

Our results demonstrate that a decrease rather than low temperature and humidity per se during the preceding three days increase the risk of influenza episodes in a cold climate.

## Background

Respiratory tract infections (RTI) are the most common infections worldwide, and hence pose a considerable economic burden to healthcare services. There is substantial evidence on the seasonal variation of respiratory morbidity and mortality, which results in increased use of health services and hospital admissions during the winter months [1]. Influenza epidemics constitute a serious public health problem associated with increased morbidity and mortality, especially in high risk populations. Worldwide, these annual epidemics result in about three to five million cases of severe illness, and about 250 000 to 500 000 deaths [2]. Seasonal variation of Influenza A and B outbreaks is a well-known phenomenon and it explains a substantial proportion of excess winter morbidity and mortality [3].

Meteorological parameters, in particular temperature and humidity, may contribute to the observed seasonal variation in the occurrence of influenza episodes. Studies from temperate and tropical climates have demonstrated that low temperature [4] and humidity increase the risk of seasonal influenza onset in the winter [5-8]. Low temperature and dry air was recently reported to increase influenza and pneumonia mortality [9]. Furthermore, experimental studies have shown that both low temperature and humidity favor the spread of influenza viruses [10-13]. Although surrounded by controversy, available scientific evidence suggests that either inhalation of cold air or cold stress cause pathophysiological responses that may contribute to an increased susceptibility to viral infections [14]. Low humidity and influenza, on the other hand, may be connected through changes in the virus stability and transmission [13].

The association between temperature, humidity and the occurrence of influenza has remained unclear. Our previous study conducted in a northern climate demonstrated that the occurrence of respiratory tract infection is associated with both low temperature and humidity [15]. However, to our knowledge; no corresponding studies examining the association between influenza virus infections, temperature and humidity have been conducted in cold climates with subfreezing temperatures. The objective of the present study was to examine the relations between temperature, humidity and the risk of influenza virus infections in a subarctic climatic zone in Northern Finland. For this purpose, we conducted a case-crossover study among young army conscripts during their military training period. Our hypothesis was that decreased daily temperature and humidity during outdoor training and associated with physical exercise would increase the risk of influenza A and B virus infection.

## Methods

### Study subjects

The study population included 892 military recruits (mean age 19.6 ± 0.8 years) from the two intake groups enrolling in military service in July 2004 and in January 2005 in the Kajaani garrison in northern Finland (64°N, 27°E). The follow-up period was from July 2004 to the end of December in 2005. The total number of men in the intake group was 1,836 in July 2004 (participation 82%) and 1,861 in January (75%). Both intake groups served part of their military training during the winter season.

### Ethics statement

The study protocol was approved by the Medical Ethics Committee of the Kainuu Central Hospital. The conscripts were described of the study, the performed assessments, possible inconvenience, the confidentiality and storing of data both in written and oral form. In addition, they were informed that participation is voluntary and that they can discontinue their participation at any time. In addition, it was clearly indicated that all potential participants who declined to take part of the study were eligible for treatment and were not disadvantaged in any other way by not participating. The conscripts provided their written consent following this information.

### Identification of cases and diagnosis of influenza A and B

This study focussed on influenza A and B cases, because they can be identified on the basis of severe symptoms which lead to a contact with medical service. Influenza A and B also have similar seasonal variation indicating identical environmental determinants of the onset. Influenza C was excluded because of milder and more variable symptoms compared with influenza A and B as well as different type of seasonal pattern which indicates different environmental causes.

Conscripts who sought medical attention for acute respiratory infections at the military primary health care clinic of the Kainuu Brigade were assessed initially by a nurse and then examined by a physician for diagnosis and treatment, if considered necessary. They were also asked to fill in a questionnaire assessing outdoor training and symptoms during the three preceding days. At that time sputum (n = 379) samples were collected. In addition, acute and convalescent serum samples were obtained (n = 520 pairs). The diagnosis of influenza was based on a positive PCR test or a significant increase in influenza A or B antibodies in paired serum samples.

Nucleic acids were extracted from 100 μl of sputum sample. Viral RNA was reverse transcribed into cDNA and influenza A and B viruses were detected by amplifying cDNA in real-time multiplex PCRs [16]. Serum samples were tested for IgG antibodies to influenza A, and B viruses by enzyme immunoassay using extracts of Madin Darby canine kidney cells (MDCK) infected with either influenza A/Beijing/353/89, or influenza B/Panama/45/90 viruses, respectively. Uninfected MDCK cells were served as control antigen. A fourfold or greater increase in antibody titers between acute- and convalescent-phase sera was considered significant.

### Exposure assessment and measures

Exposure assessment was based on meteorological data obtained from the Kajaani Airport Meteorological Station; the nearest station of the Finnish Meteorological Institute located approximately 15 km from the garrison. Average daily temperature and absolute humidity (AH) were calculated based on eight separate measurements conducted at three-hour intervals per day. In addition, an average of the three preceding days (e.g. a 72 h period) from the visit to the clinic (day 0) were calculated and used in the regression models (see below). We also separately examined the maximal changes, as well as the starting level from where temperature and AH changed. A maximum decline in temperature and AH was calculated as the largest change (maximum versus minimum) occurring in these parameters within the three day period (e.g. day −3 versus day −2, day −3 versus day −1, day −2 versus day −1 etc.). The meteorological conditions of the day the conscripts visited the clinic for influenza were not included to the analyses.

### Statistical analyses

The present study utilized a case-crossover design which is most suitable for studying relations with the following characteristics: 1) the individual exposure varies within short time intervals; 2) the disease has abrupt onset and short latency for detection; and 3) the induction period is short [17]. For ambient temperature and AH the hazard period was defined as three days preceding the visit to the clinic for a respiratory infection (and when virological samples were collected) on the basis of the estimated incubation period of 1–2 days for influenza [18]. A symmetric bidirectional selection of two reference periods shortly before and after the hazard period was utilized [19]. This means that for temperature and humidity, three-day periods seven days before and after the visit to the clinic for influenza were used in the models. This type of symmetrical selection of reference periods controls for temporal and seasonal confounding [19]. Means in temperature and AH between the hazard and reference periods were compared with one-way ANOVA. Conditional logistic regression models were used to calculate the exposure odds ratios (OR) for the hazard period compared with the reference periods. The maximal change in temperature and AH was adjusted for its initial level in the analyses. The PHREG procedure was applied using the discrete logistic model and forming a stratum for each matched set. Thus the OR represented the risk of the onset of influenza in relation to the level or change of temperature and AH. Statistical analyses were performed by SAS version 9.2. for Windows (SAS Institute, Inc.; Cary, NC).

## Results

The total number of cases was 66; 57 influenza A episodes and 9 influenza B episodes. The incidence of influenza A was 105/1000 person years and influenza B 16.5/1000 person years. The symptoms preceding the identification of influenza started on average three days before seeking medical attention. 69% (45/65) reported outdoor training, 76% (33/43) physical exercise and 72% (31/43) feeling cold during the previous three days.

**Figure 1:**
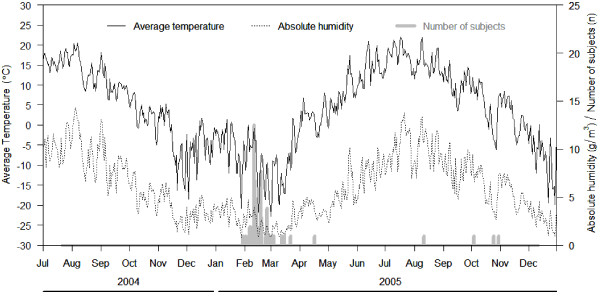
**Incidence of influenza episodes, mean daily temperature (°C) and mean daily absolute humidity (AH) (g/m**^**3**^**) during the study period.**

The daily average temperature, absolute humidity and the number of influenza episodes during the study period are presented in Figure 1. As the total number of days with episodes was 28, we utilized meteorological information from a total of 84 days. During the study period the daily average temperatures ranged from −22.8 to +22.0°C and AH from 0.8 to 13.8 g/m^3^. Most influenza cases occurred between January and March 2005 with only a few episodes occurring during the autumn months.

**Figure 2:**
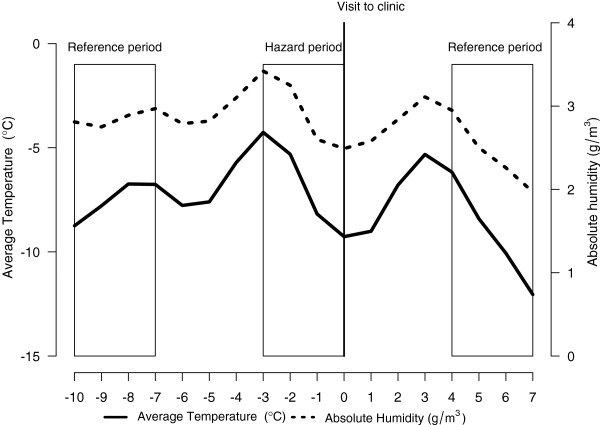
**Mean daily temperature (°C) and absolute humidity (g/m**^**3**^**) during the hazard and reference periods starting 7 days before and after the beginning of the hazard period.** Values represent means of the observed cases of influenza (n = 66). For the logistic regression analyses the mean temperature and AH was calculated from the three preceding days (day-3 to 0) of the onset of an influenza infection and similarly for the reference periods 7 days before and after the infection. A maximum decline in temperature and AH was calculated as the largest change (maximum versus minimum) occurring in these parameters within the three day period (e.g. day −3 versus day −2, day −3 versus day −1, day −2 versus day −1 etc.) and similarly for the reference periods.

Figure 2 demonstrates mean daily temperature and AH during the hazard and reference periods for the entire study population (n = 66). The figure demonstrates that the occurrence of influenza is preceded by decreases in both temperature and humidity.

Table 1 shows the means, their average before the decline, as well maximal declines in temperature and AH separately for the hazard and reference periods. The mean temperature was slightly higher during the hazard compared to the reference periods (p < 0.05). Also, the maximal decline during hazard period was greater for both temperature and humidity compared with the reference periods (p < 0.001). Of note, the temperature but not AH within the hazard period before they declined was also higher (p < 0.05) compared with the reference periods.

**Table 1:** Mean temperature and AH, their maximal declines and level preceding the decline during the hazard period (n = 66) prior to the onset of influenza episodes and during the reference periods either seven days prior or after the visit to the clinic for influenza (day 0)

**Parameter**	**Hazard period**	**Reference period (pre)**	**Reference period (post)**
Mean temperature °C	−6.76 ± 5.60	−7.51 ± 5.54	−9.17 ± 5.96^a^
Max temp decline °C	5.09 ± 3.26	1.29 ± 4.95	4.75 ± 3.79^b^
Temperature level preceding the decline	−3.5 ± 5.46	−6.58 ± 6.44	−5.19 ± 5.68^a^
Mean AH g/m^3^	3.09 ± 1.34	2.82 ± 1.67	2.57 ± 1.28
Max AH decline g/m^3^	1.18 ± 0.71	0.43 ± 0.96	0.68 ± 0.54^b^
AH level preceding the decline	3.59 ± 1.6	3.13 ± 2.02	2.81 ± 0.75

Values represent mean ± SD.

one-way ANOVA, ^a^p < 0.05, ^b^< 0.001.

Table 2 shows the odds ratios and their 95% confidence intervals (CI) for the relations between onset of influenza and the average temperature, AH and their maximal changes. The risk of contracting influenza was positively associated with mean temperature and AH. A decrease in both temperature and AH (maximal change) during the three days prior to seeking medical consultation increased the risk of influenza. According to these results, a 1°C decrease in temperature and 0.5 g decrease per m^3^ in AH increased the estimated risk by 11% (OR 1.11; 95% CI 1.03 to 1.20) and 58% [OR 1.58; 95% CI 1.28 to 1.96). There was a cluster of 13 cases during the same day and the impact of the cluster was assessed in the sensitivity analysis. The exclusion of the cluster did not substantially change the associations between temperature and humidity. However, due to the small sample size the confidence intervals are wider and associations non-significant.

**Table 2:** **Onset of influenza A and B and its association with mean values and declines in temperature (per 1°C) and humidity (0.5 g/m**^**3**^**)**

**Parameter**	**Temperature (°C) OR (95% CI)**	**Absolute humidity (g/m**^**3**^**) OR (95% CI)**
Mean	1.10 (1.02 to 1.19)	1.25 (1.05 to 1.49)
Max decline*	1.11 (1.03 to 1.20)	1.58 (1.28 to 1.96)

The odds ratios (95% confidence interval) were calculated per 1°C temperature and per 0.5 g /m^3^ absolute humidity decreases.

*adjusted for the initial level before the decline.

## Discussion

There is consistent evidence that wintertime cold temperatures increase respiratory morbidity and mortality [1,15,20,21]. We conducted a case-crossover study to assess the relations between daily temperature and humidity and the risk of influenza infections in subarctic climatic zone among military conscripts. Our results indicate that the risk of influenza is related to a decrease in both temperature and humidity, but the influenza risk in general may be reduced at very low temperatures. This could be explained by the effects of temperature and humidity on both the micro-organisms and humans. On one hand the virulence of influenza is expected to be stronger near zero than at subfreezing temperatures, but on the other hand a decrease in temperature makes airways more susceptible to the onset of respiratory infections.

### Influenza risk is reduced at very low temperatures and humidity

Our novel finding was that influenza risk is reduced at very low temperatures. This observation is supported by the fact that 74% of the influenza infections occurred at a temperature range of +5 to −10°C (and 38% between +5°C to −5°C) which is not especially cold for the climate. Also our previous study from the same population showed that the occurrence of respiratory tract infections was the highest when temperature was at or slightly below 0°C [15]. Similarly very low AH reduced the risk of influenza, which was demonstrated by a slightly higher AH during the hazard (3.09 g/m^3^) compared with the reference periods (2.82 and 2.57 g/m^3^).

Our results from subfreezing environmental conditions differ from previous experimental studies which have shown that low temperature (incubation at +21 − +24°C) increased the survival time and accelerated the transmission of the influenza virus [10,12]. Lowen [13] showed that influenza transmission increases when guinea pigs are housed at low temperature (+5°C) conditions and relates this to the effect of temperature on the physical barriers of the host. Also low humidity may improve influenza transmission due to altered function of the respiratory tract of the host [22,23] or enhanced stability of the virus particle [10-13]. Especially for conditions of low temperature and dry air the viability of influenza A is the highest when RH is below 50% [24]. Furthermore, in conditions of low absolute humidity (such as in a cold climate) droplet nuclei are small sized and remain airborne for extended periods of times increasing the opportunity for transmission of pathogens they carry and favouring the spread of influenza [25-28].

The reason why we observed a reduced risk of influenza at low temperature and AH could indicate that the optimal temperature and humidity for the viability, transmission and replication of influenza occurs at higher environmental temperatures and humidity levels. This assumption is supported by a recent epidemiologic study from US which showed that influenza mortality increased when AH decreased below 6 g/kg and peaked when temperature was −1.1°C [29]. Though, this study used monthly means in their data analyses instead of daily averages used in the present study.

### A decline in temperature and humidity increases the risk of influenza

We observed that a decrease in temperature and AH increased the risk of influenza. For temperature the risk was associated with a higher initial levels before the decline. This seemingly contradictory finding could be explained by a novel idea: we should consider the effects of temperature on the viral agent separately from that of the host. Higher temperatures approaching zero degrees may favour transmission and survival of the virus itself, but a decline in temperature and humidity may make the host more susceptible through body cooling and/or drying of the respiratory tract.

A sudden decrease in temperature and humidity could be related to an increased experience of cold stress and altered airway function increasing the susceptibility to viral agents. Military wintertime training is associated with significant cold exposure and heavy physical exercise which could result in aggravated airway cooling and functional changes of the airway epithelia favouring influenza virulence and other respiratory tract infections [15]. It has been suggested that even cooling of the body surface could elicit a reflex vasoconstriction in the nose and upper airways and inhibit the respiratory defence and convert an asymptomatic subclinical viral infection into a symptomatic clinical infection [14]. Alternatively, breathing cold air causes cooling of the upper respiratory tract and results in vasoconstriction [30]. Also inhaling larger volumes of air with low AH causes drying of the mucosal membrane [22], which can even lead to epithelial damage [23]. These effects could depress the ciliary movement in the respiratory tract and increase the susceptibility to infections. Although no conclusive evidence exists, the seasonal variation in immune responses could further contribute to host susceptibility to infections [31]. Our novel finding suggests that a combination of relatively warmer temperature and higher humidity followed by a sudden decline in these meteorological parameters have the strongest impact on the risk of influenza. It should be remembered that both temperature and humidity are interconnected for which it is not meaningful to evaluate their independent roles in the present study.

### Previous epidemiological studies on meteorological parameters and influenza

To our knowledge none of the previous research assessing influenza morbidity originates from very cold climates, such as this study. However, the association between temperature and humidity and the occurrence of influenza has been demonstrated in epidemiological studies examining onset [4-8] and outcomes, such as mortality [3,7,9,29].

An increased influenza risk with low air temperatures has been observed in temperate [4,7,9] or tropical countries [5,32]. Furthermore, low humidity [7,9], or its decrease [8] increases the occurrence of influenza. A recent study demonstrated that low wintertime outdoor AH is also reflected as reduced indoor humidity and could predict increased influenza virus survival [33]. An inverse correlation between AH and temperature was shown at the onset of infection in a study modeling influenza-like illness and weather factors from three European countries [34]. Mortality studies from moderate climates, such as the US have demonstrated that low temperature [9] and humidity [7,9] during the prior few weeks were associated with increased wintertime influenza-related mortality. Especially low AH is suggested to explain a larger portion of the observed mortality [6,29]. These studies support our finding that both temperature and AH are associated with the risk of influenza. However, a direct comparison of the results between these studies is not possible because of the different modes of influenza transmission between tropical, moderate or cold climates [35].

The significance of meteorological parameters for explaining the seasonality of influenza is under debate [36-38] and it has been suggested that temperature and humidity could be accessory or modulating factors for example in the timing of the onset of an influenza epidemic [3]. In addition, the importance of either temperature or humidity on the occurrence of influenza is being discussed [6,29,35]. Due to the close dependence of these physical quantities it may be difficult, or not even meaningful, to examine their independent roles. Potential other explanatory factors for the seasonality of influenza include alterations in host susceptibility (e.g. immunity), viral mutations, as well as the effects of weather on social contact patterns [39] during the wintertime which enhances influenza spread.

### Strengths and limitations of the study

We used a case-crossover design which eliminates confounding by stable individual characteristics and by determinants of influenza risk including crowding and annually occurring respiratory infection epidemics. The fact that, compared to the general population, military conscripts are more frequently exposed to low temperatures for prolonged periods, gives additional strength to this study. In addition, physical exercise may aggravate respiratory cooling and the onset of influenza. Military service in Finland is mandatory for young men, and conscripts thus represent the ordinary population of this age group. As study limitations, we were not able to control for person-to-person transmission, which is likely to occur in a military setting and would facilitate the spread of the virus. In principle, time progression could also be analysed, by using calendar time as an effect modifier, and could provide additional information. However, unfortunately the amount of data was not sufficient for such analyses. Furthermore, a non-zero reinfection probability would affect the transmission dynamics, but its detailed analysis was not possible with the current data. Clustering of influenza cases to a relatively narrow temperature and AH range could reduce the width of the confidence intervals, but would not likely affect the point estimates of the meteorological variables on influenza. However, we conducted a sensitivity analysis which showed a similar association (not significant) after excluding the possible effect of an influenza epidemic. Yet, larger samples from equal climatic conditions and follow-up of multiple influenza seasons would be useful to strengthen our findings. One methodological limitation was due to difficulties in the collection of sputum samples which may lead to underdiagnoses of cases. Given the gap in knowledge, our results provided a good starting point and a priori hypothesis for further studies.

## Conclusions

Wintertime influenza epidemics can cause serious public health and economic problems including worker absenteeism and productivity losses, burdening of health care services due to hospitalisation, and in the worst case, deaths. An improved understanding of influenza virus transmission is of importance in order to enhance the accuracy of surveillance systems, to have more precise predictions on influenza epidemics and pandemics in the future, and eventually to develop better disease-control intervention strategies.

In conclusion, our study suggests that a decline in temperature and humidity is associated with the occurrence of influenza infection episodes in young healthy men during wintertime in a subarctic climate. However, very low temperatures and absolute humidity may even reduce the occurrence of influenza infections. Further studies are needed to confirm causality and establish threshold values between temperature, humidity and influenza infections. Our findings are important from a public health perspective and should be considered when planning appropriate cold risk management strategies.

## Abbreviations

AH: Absolute humidity; OR: Odds ratio; CI: Confidence interval.

## Competing interests

The author(s) declare that they have no competing interests.

## Authors’ contributions

KJ participated in the analyzing and interpretation of the study, drafted the manuscript, and is the principal author of the manuscript. AS participated in the analyzing and interpretation of the study and participated in writing the manuscript (special expertise: microbiology). JJ participated in the analysis and interpretation of data (special expertise: statistical analyses) and writing of the manuscript. RJ participated in the conception and design of the study, implemented the experimental protocol and collection of data, participated in the analysis and interpretation of data and in writing the manuscript (special expertise: otorhinolaryngology). JK participated in the analysis and interpretation of data (special expertise: virological analyses) and writing of the manuscript. OV participated in the analysis and interpretation of data (special expertise: microbiology) and writing of the manuscript. TZ conducted the virological analyses, participated in the analysis and interpretation of data (special expertise: virology) and writing of the manuscript. ES conducted the virological analyses, participated in the analysis and interpretation of data (special expertise: virology) and writing of the manuscript. JJK participated in the analysis and interpretation of data (special expertise: environmental health, case-crossover analysis) and writing of the manuscript. TMI participated in the analysis and interpretation of data (special expertise: environmental effects on human health and performance) and writing of the manuscript. All authors have approved the final version of the manuscript.
